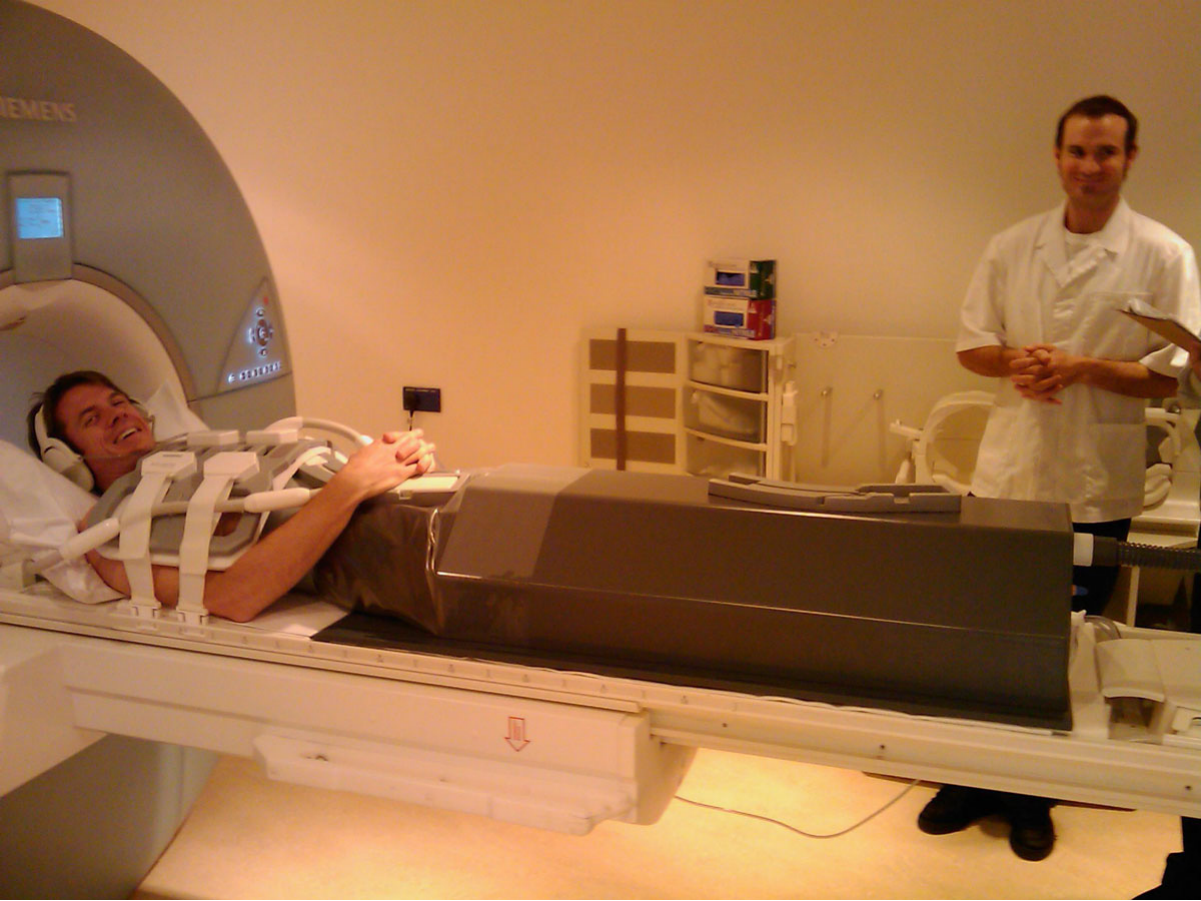# Cardiac pre-load alteration with MRI-compatible lower body suction device

**DOI:** 10.1186/1532-429X-17-S1-Q113

**Published:** 2015-02-03

**Authors:** Gergely V Szantho, Chris B Lawton, Stephen Lyen, Chiara Bucciarelli-Ducci, Nathan E Manghat, Mark S Turner, Michael P Frenneaux, Mark Hamilton

**Affiliations:** 1 Bristol Heart Institute, Bristol, UK; 2 University of Bristol, Bristol, UK; 3 University of Aberdeen, Aberdeen, UK

## Background

Our group studies circulatory causes of exercise limitation in congenital heart patients with dilated right ventricle, but normal resting left ventricular ejection fraction. Cardiac MRI is the gold standard non-invasive tool for measuring heart chamber volume and blood vessel flow. We designed an MRI-compatible lower body negative pressure (LBNP) device (*Image*) in order to study the effect of pre-load reduction on the heart with MRI*.* This is a pilot study to establish our methodology.

## Methods

We measured ventricular volumes from standard contiguous short axis stack of gated SSFP cines (slice thickness 8mm), and great vessel flows just above the sino-tubular junction from standard phase contrast flow imaging at rest and at different levels of lower body vacuum (-5, -10 and -20mmHg) in nine healthy volunteers. We compared measurements taken at rest and different levels of vacuum with students' paired t-test.

## Results

We found that end-diastolic volume (EDV) and stroke volume (SV) progressively decreased with increasing lower body vacuum, in both ventricles. The difference between rest and -20mmHg LBNP were as follows: LVEDV 160.64+-28.18ml vs 145.02+-25.61ml (p<0.0005), LVSV 98.44+-15.88ml vs 84.9+-12.38ml (p<0.005), RVEDV 174.81+-46.01ml vs 154.1+-34.65ml (p<0.01), RVSV 100.11+-18.34ml vs 89.51+-17.62ml (p<0.1 ns).

Peak flow rate (PFR) and stroke volume measured with flow imaging also decreased both in the ascending aorta and the main pulmonary artery (MPA). The difference between rest and -20mmHg LBNP were as follows: Aortic SV 88.11+-16.72ml vs 72.8+-13.85ml (p<0.001), Aortic PFR 441.5+-75.84ml/s vs 387.47+-56.01ml/s (p<0.002), MPA SV 105.25+-19.86ml vs 84.42+-20.7ml (p<0.00002), MPA PFR 442.48+-84.38ml/s vs 375.22+-6974ml/s (p<0.0002).

Heart rate (HR) increased slightly from rest at -20mmHg LBNP: 63.58+-10.49bpm vs 69.43+-11.76bpm (p<0.01).

Interestingly RVEDV, RVSV and MPA SV were already significantly reduced at -5mmHg LBNP (165.9+-43.28ml p<0.002, 89.34+-15.42ml p<0.005, 95.99+-15.75 p<0.05,respectively); and most of the other parameters changed significantly already at -10mmHg, which will be further disussed.

Blood pressure did not change significantly, and our volunteers did not indicate any discomfort.

## Conclusions

Our pilot study shows that our MRI compatible LBNP device is safe, exerts adjustable and non-invasive pre-load reduction, and the physiological cardiac response to it is well detectable with cardiac MRI.

## Funding

Funded by the general research budget of our Institution.

**Figure 1:**